# Heat shock protein 90 inhibitors repurposed against *Entamoeba histolytica*

**DOI:** 10.3389/fmicb.2015.00368

**Published:** 2015-04-28

**Authors:** Dea Shahinas, Anjan Debnath, Christan Benedict, James H. McKerrow, Dylan R. Pillai

**Affiliations:** ^1^Department of Laboratory Medicine and Pathobiology, University of TorontoToronto, ON, Canada; ^2^Skaggs School of Pharmacy and Pharmaceutical Sciences, University of CaliforniaSan Diego, La Jolla, CA, USA; ^3^Department of Pathology and Laboratory Medicine, University of CalgaryCalgary, AB, Canada

**Keywords:** EhHsp90 inhibitors, rifabutin, rutilantin, cetylpyridinium chloride, pararosaniline pamoate, gentian violet

## Abstract

Hsp90 is an essential chaperone responsible for trafficking a vast array of client proteins, which are substrates that Hsp90 regulates in eukaryotic cells under stress conditions. The ATP-binding N-terminal domain of Hsp90 (also known as a GHKL type ATPase domain) can serve as a specific drug target, because sufficient structural diversity in the ATP-binding pocket of Hsp90 allows for ortholog selectivity of Hsp90 inhibitors. The primary objective of this study is to identify inhibitors specific for the ATP-binding domain of *Entamoeba histolytica* Hsp90 (EhHsp90). An additional aim, using a combination of site-directed mutagenesis and a protein *in vitro* assay, is to show that the antiparasitic activity of Hsp90 inhibitors is dependent on specific residues within the ATP-binding domain. Here, we tested the activity of 43 inhibitors of Hsp90 that we previously identified using a high-throughput screen. Of the 43 compounds tested, 19 competed for binding of the EhHsp90 ATP-binding domain. Five out of the 19 EhHsp90 protein hits demonstrated activity against *E. histolytica in vitro* culture: rifabutin, rutilantin, cetylpyridinium chloride, pararosaniline pamoate and gentian violet. These five top *E. histolytica* Hsp90 inhibitors showed 30–100% inhibition of *E. histolytica* in culture in the micromolar range. These data suggest that *E. histolytica*-specific Hsp90 inhibitors are possible to identify and provide important lead compounds for the development of novel antiamebic drugs.

## Introduction

*Entamoeba histolytica* is a protozoan intestinal parasite that causes amebiasis worldwide (Walsh, 1986), Amebiasis presents as diarrhea in humans causing 50 million cases of invasive disease and 70–100 thousand deaths worldwide (Stanley, 2003; Ralston and Petri, 2011b). Current therapy relies solely on metronidazole and is complicated by resistance and adverse neurotoxic, mutagenic and carcinogenic effects (Freeman et al., 1997; Kapoor et al., 1999; Bendesky et al., 2002). Considering the prevalence of amebiasis and the lack of other therapeutic options, it is of paramount importance to search for other effective, better-tolerated antiamebic drugs. To this end, we propose the discovery of new antiamebic drugs by targeting heat shock protein 90 (Hsp90), one of the best-studied members of the heat shock protein (HSP) family.

Stress inducible cytosolic Hsp90 exists in the form of a multi-chaperone complex and is essential for helping client proteins to fold (Li and Buchner, 2013). As such, it is essential for normal eukaryotic growth and development (Li and Buchner, 2013). The existing model suggests that the stress inducible isoform of cytosolic Hsp90 serves as a buffer of phenotypic variation and potentiator of drug resistance by preventing cellular toxicity caused by misfolded and aggregated proteins in response to heat shock or pharmacological stress (Cowen and Lindquist, 2005; Cowen et al., 2009; Marubayashi et al., 2010). Many Hsp90 clients are essential cellular proteins with pathogenic functions that render the inhibition of the Hsp90 pathway lethal in cells undergoing pathogenic, pharmacological or heat-shock stress, but not in normal cells (Cowen and Lindquist, 2005; Chiosis et al., 2006; Cerchietti et al., 2009, 2010; Cowen et al., 2009; Taldone et al., 2010). Pharmacologic inhibition of Hsp90 effectively results in lethality in abnormal cells such as infected or transformed cells by disruption of the broad spectrum of Hsp90 interactions and signaling pathways (Jhaveri et al., 2011; Usmani and Chiosis, 2011). As such, this inhibition provides specific anti-disease effects and a decreased likelihood for developing resistance.

In particular, inhibition of this ATPase activity at the N-terminal ATP-binding domain is an effective approach for blocking its function and interaction with client proteins (Jhaveri et al., 2011; Usmani and Chiosis, 2011). Significant similarity exists at the ATP-binding domain between other eukaryotic stress-inducible Hsp90s and parasite Hsp90s such as *Plasmodium falciparum* Hsp90 (PfHsp90) and *E. histolytica* Hsp90 (EhHsp90) (Banumathy et al., 2003; Pavithra et al., 2004, 2007; Acharya et al., 2007; Kumar et al., 2007; Pallavi et al., 2010). In addition, the pocket architecture of the orthologous ATP binding domains of stress inducible Hsp90 also contains unique residues that can be selectively targeted by inhibitors (Wider et al., 2009; Corbett and Berger, 2010; Pallavi et al., 2010). Such unique residues may be involved in ligand binding, but do not participate in the catalytic function of this domain, because residues involved in catalysis are essential for the ATPase function of this domain and are therefore conserved (Wider et al., 2009; Corbett and Berger, 2010; Pallavi et al., 2010). For example, crystal structures of human and *P. falciparum* Hsp90 N-terminal domains (PDB ID: 2FWZ and 3K60, respectively) reveal that PfHsp90 Met84 adopts a different side-chain rotamer than human Met98, altering the shape of the “ceiling” of the binding pocket (Corbett and Berger, 2010). Ser52, Lys112 and Val186 of human (Hs) Hsp90 are replaced by Ala38, Arg98 and Ile173 in PfHsp90 (Corbett and Berger, 2010). Even though these residues are not involved in ATP hydrolysis (Corbett and Berger, 2010), in general, these differences in pocket architecture suggest that the PfHsp90 ATP-binding domain is slightly more hydrophobic, constricted, and basic relative to the human ortholog (Corbett and Berger, 2010).

We hypothesize that targeting of cytosolic-inducible Hsp90 is a viable strategy for drug discovery as this protein is essential in most eukaryotes studied to date e.g., *Drosophila melanogaster* (Bandura et al., 2013), *Caenorhabditis elegans* (Inoue et al., 2003), *P. falciparum* (Banumathy et al., 2003). In addition, conservation of the N-terminus ATP binding domain suggests that this domain is under selection pressure to be conserved so that it may provide the energy needed to fold client proteins. Due to both this conservation, and presence of unique residues that may confer selectivity, repurposing of previously identified Hsp90 inhibitors is an attractive strategy and opportunity to capitalize on existing safety and pharmacokinetic data. Therefore, we tested the effect of 43 previously identified HsHsp90 and *P. falciparum* (PfHsp90) (Shahinas et al., 2010) inhibitors for their ability to inhibit EhHsp90. The objectives of this study were: (1) to biochemically characterize and identify the unique residues in the ATP binding pocket of HsHsp90 vs. EhHsp90; (2) to explore the selectivity of binding of these inhibitors to EhHsp90, PfHsp90 and HsHsp90 as well as to the site-directed mutants of each of these domains (3) to assess the effect of selective inhibitors on *E. histolytica* in cell culture.

## Materials and methods

### Cloning and protein purification

A *P. falciparum* Hsp90 ATP-binding domain construct was amplified from genomic DNA harvested from the intra-erythrocytic life cycle of *P. falciparum* strain 3D7 obtained from the MR4 Malaria Research and Reference Resource Center (Forward primer: 5′ CGCCGGCGCCATATGAGTTTTCCAAG 3′ Reverse primer 5′ CGCCGGCGCGGATCCTAAATTCATTAAACT 3′) and was cloned into the pET28b vector (Novagen). The *E. histolytica* Hsp90 ATP-binding domain was also amplified from genomic DNA extracted from a frozen *E. histolytica* (strain HM1:IMSS) culture obtained from the American Type Culture Collection (ATCC) (Forward primer: 5′CCGGGATCCATGGGAAATAGAAAA3 ′) (Reverse primer: 5′GCGCGGTTCGAAATATTGAATAAATTC 3′) and was cloned into the pET28a vector (Novagen).

The clones were expressed in *E. coli* Bl21 (DE3) Codon Plus cells (Agilent Technologies) grown in terrific broth (12 g tryptone, 24 g yeast extract, 17 mM KH_2_PO_4_, 72 mM K_2_HPO_4_ and 4 mL glycerol per liter broth), and induced with 0.4 mM Isopropyl β-D-1-thiogalactopyranoside (IPTG) overnight at 24°C. These *E. coli* cells were harvested by centrifugation (5000 rpm for 10 min), resuspended in lysis buffer (20 mM 4- (2-hydroxyethyl)-1-piperazineethanesulfonic acid (HEPES), pH 7.5, 10% glycerol, 20 mM imidazole, 500 mM NaCl, 0.5% nonyl phenoxypolyethoxylethanol NP-40 surfactant) and supplemented with 100× bacterial protease inhibitor cocktail (Sigma). Cells were lysed by 5 times 45 s rounds (separated by a pause of 45 s) of sonication. The cell debris was removed by centrifugation (14,000 rpm for 30 min), and the protein was purified using nickel-nitrilotriacetic acid (Ni-NTA) resin (QIAGEN). Tobacco etch virus (TEV) protease was added at a ratio of 1:50 TEV protease in dialysis buffer [20 mM HEPES, pH 7.5, 100 mM NaCl, 5 mM MgCl_2_, and 0.01 mM 4,40-dianalino-1,10-binaphthyl-5,50-disulfonic acid dipotassium salt (bis-ANS)] and incubated overnight at 4°C. This mixture was washed over a Ni-NTA column to remove the TEV protease and cleaved polyhistidine tags from the purified protein. The proteins were concentrated to ~10 mg/mL using Amicon® Ultra centrifugal filters for protein concentration (Millipore) and centrifugation at 2800 rpm. The same conditions were used for the expression and purification of all the proteins used in this study. The HsHsp90 ATP binding domain pET15b (Novagen) clone was kindly provided by Dr. Daniel Gewirth (Hauptman-Woodward Medical Research Institute).

### Site-directed mutagenesis

Site-directed mutants were generated using site-specific primers (Supplementary File 2) that target unique residues in the ATP binding pockets of HsHsp90, PfHsp90, and EhHsp90 proteins. The site directed mutagenesis procedure was followed as per instructions of the QuickChange site-directed mutagenesis kit (Agilent Technologies, Santa Clara, CA) with some modifications. Briefly, the mutant plasmid was amplified using the proofreading enzyme *Pfx* (Invitrogen, Carlsbad, CA). Denaturation of the initial template was allowed to take place for 3 min at 95°C. After the initial denaturation, 14 cycles of denaturation (95°C), annealing (58°C) and elongation (68°C) took place. The nascent template was digested by the *Dpn1* enzyme (37°C for 1 h), which digests methylated DNA that has been replicated inside *E. coli* Dh5-alpha bacteria (Invitrogen), which were used for the plasmid purification in this case. The undigested (amplified) plasmids were transformed in ultracompetent *E. coli* XL-Gold 10 cells (Agilent Technologies, Santa Clara, CA). The plasmids were purified using the Qiagen Miniprep kit (Qiagen, Germantown, MD). These plasmids were screened for the presence of the mutation using Sanger sequencing (Applied Biosystems 3130*xl*, Carlsbad, CA) after amplification of the Hsp90 ATP binding domain gene using standard T7 primers and annealing conditions (Novagen, Madison, WI).

### 4,40-dianalino-1,10-binaphthyl-5,50-disulfonic acid dipotassium salt (bis-ANS) binding assay with the PfHsp90 ATP-binding domain

By optimization of a previously established technique (Wassenberg et al., 2000), the fluorescent probe 4,40-dianalino-1,10-binaphthyl-5,50-disulfonicacid dipotassium salt (bis-ANS, Sigma-Aldrich) was used to demonstrate nucleotide binding to the ATP-binding domain of PfHsp90 (Shahinas et al., 2010). Recombinant purified protein (final protein concentration 1 μM) was pre-incubated for 45 min at 37°C with no drug or in the presence of 200 nL of drug to a final concentration of 100 nM (Spectrum and Lopac libraries) and 50 nM (Prestwick library). bis-ANS was then added to a final concentration of 5 μM in binding buffer [20 mM tris(hydroxymethyl) aminomethane(Tris) pH 7.5, 10 mM MgCl_2_, 50 mM KCl] in a final volume of 20 μL and incubated at 37°C for 30 min. In order to facilitate the high throughput screening of 4000 compounds, all these volumes were optimized for robotic handling. Drug distribution was carried out using a pintool that can accurately dispense 200 nL volumes. Fluorescence emission data were collected on an EnVision fluorescent monochromator spectrophotometer (Perkin-Elmer Life Sciences). Excitation wavelength for bis-ANS was set at 372 nm, and emission was captured at 490 nm. All chemical compounds had 99% purity by high performance liquid chromatography (HPLC).

### *Entamoeba histolytica* culture experiments

*E. histolytica* trophozoites (HM1:IMSS strain from ATCC) were maintained in TYI-S-33 medium (20 g casein digest, 10 g yeast extract, 10 g glucose, 2 g sodium chloride, 1 g L-cysteine HCl, 0.2 g ascorbic acid, 1 g K_2_HPO_4_, 0.6 g KH_2_PO_4_, 28.75 mg ferric nitrate per liter) that was supplemented with penicillin (100 U/mL), streptomycin (100 μg/mL), and 10% adult bovine serum, under axenic conditions according to the methods of Diamond (Diamond et al., 1978; Debnath et al., 2004, 2005). All of the experiments were conducted with trophozoites that had been harvested during the logarithmic phase of growth. The logarithmic phase of growth was determined by counting the cells using the particle counter (Beckman Coulter) and the cells were maintained in this growth phase by routine passage every 2 days. Compounds were tested for antiparasitic activity using an ATP-bioluminescence based screen for cell growth and survival (Debnath et al., 2012). Assay plates were inoculated with trophozoites (5 × 10^3^ parasites/well) and incubated in the GasPak™ EZ Anaerobe Gas Generating Pouch Systems (VWR, West Chester, PA) to maintain anaerobic conditions throughout the incubation period. The assays were performed in triplicate using the CellTiter-Glo Luminescent Cell Viability Assay (Promega). Metronidazole was used as a positive control because it is the current treatment for *E. histolytica* infection.

## Results

### Homology modeling reveals ortholog-specific residues in the N-terminal ATP-binding domain of Hsp90

**Figure 1:**
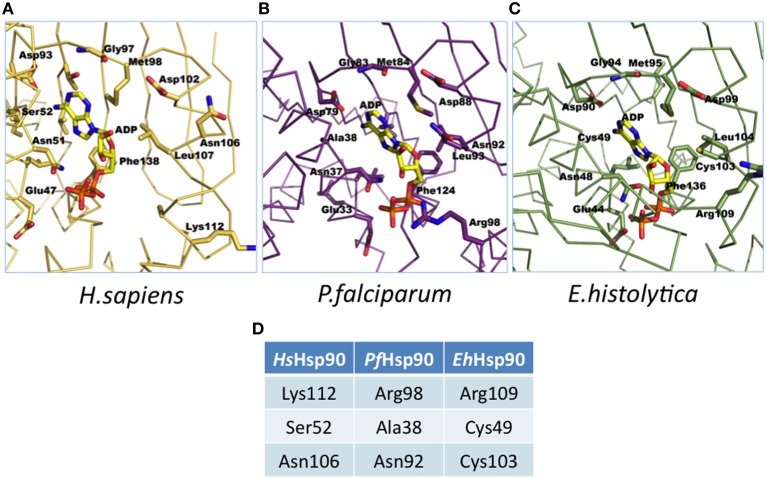
**Illustration of ATP binding pocket differences. (A)** Molecular model of adenosine diphosphate (ADP) in the adenosine triphosphate (ATP) binding pocket of human (2FWZ) Hsp90 (HsHsp90). **(B)** Molecular model of ADP in the ATP binding pocket of *P. falciparum* (3K60) Hsp90 (*Pf*Hsp90). **(C)** Molecular model of ADP in the ATP binding pocket of *E. histolytica* Hsp90 (EhHsp90) (based on PfHsp90 template: 3K60). (**D**) Summary of the unique residues in the ATP binding pockets of HsHsp90, PfHsp90 and EhHsp90. Please note that these residues are at conserved positions in the 3D model. These residues were mutated by site-directed mutagenesis to examine alterations in binding specificity.

Homology modeling studies using the crystal structures of PfHsp90 (PDB accession no. 3K60) and HsHsp90 (PDB accession no. 2FWZ) and the homology model of EhHsp90 ATP-binding domain suggest that although the pocket is overall very well conserved, there are three corresponding positions that contain unique residues in the three structures (Figure 1). Ser52, Asn106, and Lys112 in HsHsp90 correspond to Ala38, Asn92, and Arg98 in PfHsp90 and Cys49, Cys103 and Arg109 in EhHsp90. This finding suggests that the ATP-binding pocket of EhHsp90 may not be as constricted as that of PfHsp90 but it is more hydrophobic than that of PfHsp90 and HsHsp90.

### Biochemical screen with recombinant EhHsp90 ATP-binding domain

**Figure 2:**
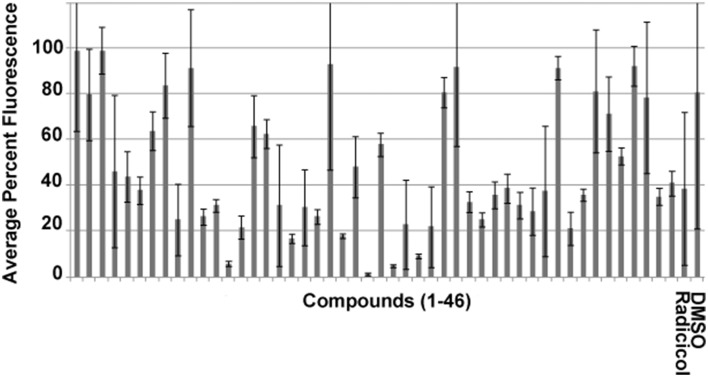
**Competitive binding assay using the fluorescent probe bis-ANS for the identification of EhHsp90 inhibitors at a final concentration of 2.5 μM**. The error bars represent standard deviation of duplicate readings. Inhibition is defined as >70% reduction of fluorescence in this assay. Compounds 1–43 are listed in Supplementary File 1. Please note that 48 compounds are shown, of which five could not be obtained any longer from the manufacturer. Therefore, the manuscript focuses on the rest 43 compounds, which were used for downstream assays.

We have previously reported the results of an initial screen vs. HsHsp90 and PfHsp90 based on competitive inhibition of bis-ANS binding with 4000 compounds consisting of natural compounds [Spectrum], FDA approved drugs [Prestwick], and pharmacologically active compounds [Lopac] (Shahinas et al., 2010). Forty-three compounds were identified that caused a reduction of >70% in fluorescence against HsHsp90 and PfHsp90 ATP binding domains, suggesting competitive inhibition of ATP-binding (Shahinas et al., 2010). This threshold of >70% fluorescence inhibition was set based on Hsp90 inhibition by radicicol, a well-known cross-species Hsp90 inhibitor (Schulte et al., 1998, 1999). Screening of these 43 inhibitors against the EhHsp90 ATP-binding domain showed that 19 of them competitively inhibit this domain by >70% (Figure 2).

### Site-directed mutagenesis of unique residues in orthologous Hsp90 ATP-binding domains affects inhibitor selectivity

**Figure 3:**
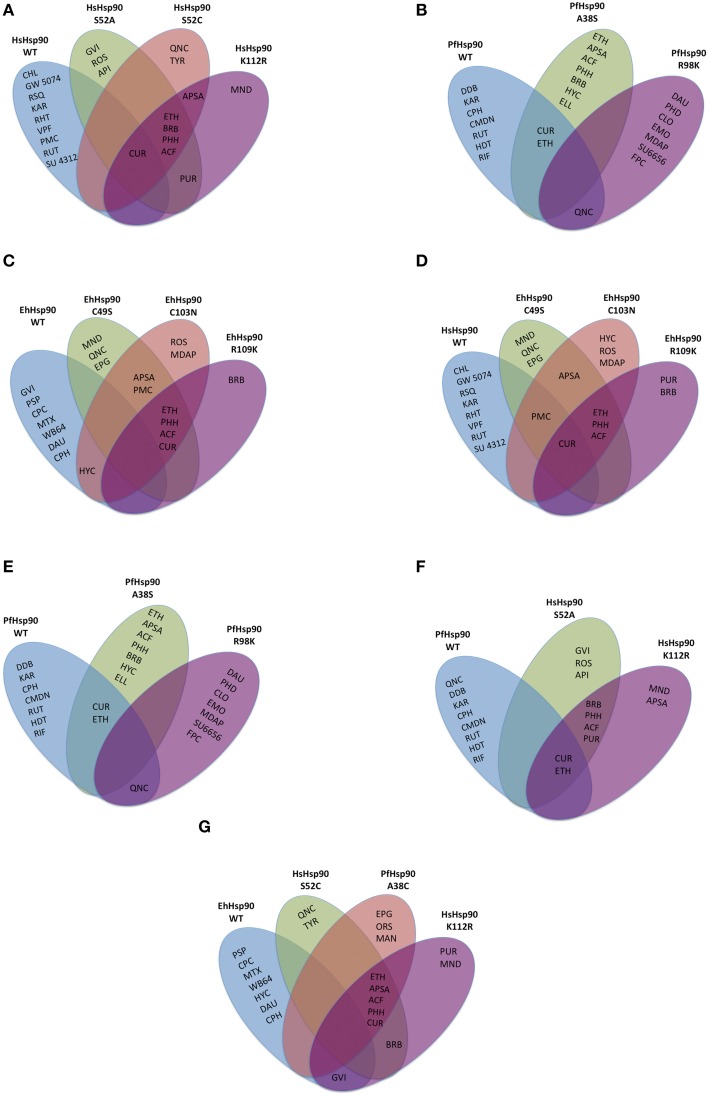
**Drug binding specificity of the Hsp90 ATP binding domains and their mutants**. Top 10 inhibitors are shown for each of the wild-type (WT) or site-directed mutant ATP binding domains. Any inhibitors in overlapping ovals are not selective. Inhibition was evaluated by competition for bis-ANS binding. Top 10 inhibitors of the HsHsp90 **(A)**, PfHsp90 **(B)**, EhHsp90 **(C)** ATP binding domains and the mutants generated in each domain, respectively. **(D)** Top 10 inhibitors of HsHsp90 and mutants that mimic HsHsp90 residues at the corresponding positions (1). **(E)** Top 10 inhibitors of HsHsp90 and mutants that mimic HsHsp90 residues at the corresponding positions (2). **(F)** Top 10 inhibitors of PfHsp90 and mutants that mimic PfHsp90 residues at the corresponding positions. **(G)** Top 10 inhibitors of EhHsp90 and mutants that mimic EhHsp90 residues at the corresponding positions. The abbreviations for the drugs displayed in this figure are listed in Supplementary File 1.

To determine the importance of the three unique positions tabulated in Figure 1D, site directed mutants were generated at each of those sites in the corresponding vectors. The proteins were expressed and the affinity of each mutant for any of the 43 compounds was determined based on competitive inhibition of bis-ANS binding using the same conditions as for the wild-type proteins. Even though, the expression level and yield varied among the different mutants, equal concentration of each protein was used to test competitive inhibition with bis-ANS. The top 10 inhibitors for the wild-type (WT) protein and each mutant are shown relative to each other in the Venn diagrams of Figure 3. Venn diagrams show common inhibitors that compete for binding (overlapping ellipses) and unique inhibitors that compete for binding of this ATP binding domain (where no overlap is observed). It is important to note that most of these residues confer selectivity of binding for the 43 inhibitors tested. There are three inhibitors among the 43 tested that show consistent non-selective high inhibition: quinacrine (QNC), curcumin (CUR), and ethaverine hydrochloride (ETH). The abbreviations used for the drugs shown in Figure 3 are listed in Supplementary File 1.

### EhHsp90 inhibitor effect on cell culture

**Figure 4:**
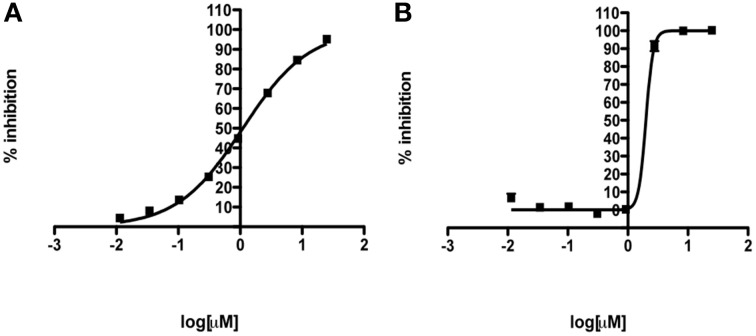
**Anti-amebic activity of (A) pararosaniline pamoate (IC50 = 1.1 μM) and (B) rutilantin (IC50 = 2.0 μM) using a standard cell-based assay**. Mean IC_50_ curves are shown. Error bars represent standard deviation of triplicate samples.

The hits of the protein screen were next tested against a standard lab strain of *E. histolytica* HM1:IMSS *in vitro*. Five out of the 19 EhHsp90 protein hits inhibited *E.histolytica* growth (replication of trophozoites) in the range of 30–100% at the concentration of 25 μM (Table 1). These five compounds were: rifabutin, rutilantin, cetylpyridinium chloride, pararosaniline pamoate, and gentian violet. Based on the site directed mutagenesis experiment outlined above, rifabutin showed the highest inhibition against PfHsp90 and displayed amino acid preferences for A38, N92, and R98. Rutilantin was present in the top 10 inhibitors of the HsHsp90 and PfHsp90 constructs and displayed preference for N106 in HsHsp90 and N92 in the corresponding position of PfHsp90. Pararosaniline pamoate and gentian violet showed highest inhibition for the EhHsp90 construct and displayed preference of binding as assessed by competitive inhibition for C49, C103, and R109. These results are summarized in Table 2. From these five compounds, it was possible to obtain titration curves for pararosaniline pamoate and rutilantin with IC50s of 1.1 μM and 2 μM, respectively (Figure 4).

**Table 1:** **Summary of inhibition results for the EhHsp90 protein screen and the effect of the inhibitors on *E. histolytica* culture**.

**Compound**	**Percent inhibition of fluorescence in protein screen at 2.5 μM (%)**	**Percent inhibition of *E. histolytica* growth at 25 μM (%)**
GW5074	70.5	–
Sanguinarine sulfate	71.3	–
Rifabutin	73.7	30
Bilirubin	73.7	–
Clofazimine	73.7	–
Purpurin	75.0	–
Tyrphostin AG 538	75.0	–
Rutilantin	77.1	100
Rifaximin	78.2	–
Berberine chloride	78.3	–
Chlorophyllide	78.9	–
Amodiaquindihydrochloride dihydrate	82.1	–
Daunorubicin hydrochloride	83.2	–
WB 64 (Malachite Green)	84.9	–
Hycanthone	90.9	–
Mitoxantronedihydrochloride	95.3	–
Cetylpyridinium chloride	95.9	42
Pararosanilinepamoate	99.0	81
Gentian Violet	99.3	35

**Table 2:** **Summary of biochemical specificity results for inhibitors of *E.histolytica* in culture**.

**Drug**	**Chemical structure**	**Highest % inhibtion**	**Amino acid prefernce**
Rifabutin (RIF)	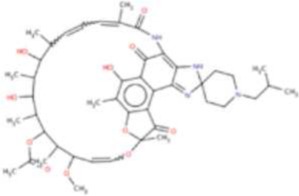	PfHsp90	PfHsp90: A38 + N92 + R98
Rutilantin (RUT)	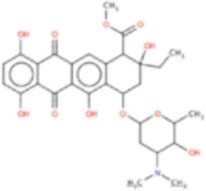	HsHsp90 & PfHsp90	HsHsp90: N106 PfHsp90: N92
Cetylpyridinium chloride (CPC)	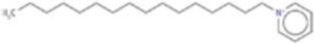	EhHsp90	EhHsp90: C49 + C103 + R109
Pararosaniline pamoate (PSP)	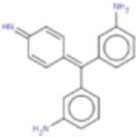	EhHsp90	EhHsp90: C49 + C103 + R109
Gentian Violet (GVI)	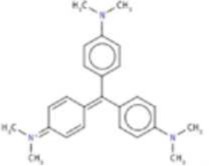	EhHsp90	EhHsp90: C49 + C103 + R109 HsHsp90: S52A or Kll2R

*The chemical structures shown here have been adapted from ChemDB (Pavithra et al., 2007; Corbett and Berger, 2010)*.

## Discussion

The discovery of five compounds that target HSP90 and inhibit *E. histolytica* growth has significant implications for antiparasitic drug development. Amebiasis caused by *E. hisolytica* is responsible for 35–50 million cases of symptomatic disease and about 100,000 deaths per year (Ralston and Petri, 2011b). Parasite cysts are transmitted through contaminated food and water (Stanley, 2003). Currently, nitroimidazoles (metronidazole) are prescribed for treatment, but despite toxic side effects, cure is not reached in 40–60% of the patients (Ralston and Petri, 2011b). In addition, novel drugs for *E. histolytica* are urgently needed as the approved therapy (metronidazole) may result in clinical treatment failure due to resistance.

Infection occurs after ingestion of cysts and may lead to liver abscess (Ralston and Petri, 2011a,b). Parasite excystation in the small intestine produces eight trophozoites per cyst, which colonize the large intestine, existing both in the lumen and attached to mucus and epithelial cells (Petri et al., 2002). Not all strains have the same virulence and are capable of causing liver abscess (Ralston and Petri, 2011b). Chaperoning of virulence factors by stress-inducible Hsp90 is likely to be important. Therefore, inhibition of Hsp90 ATP binding is a compelling strategy for antiparasitic drug design.

Our recombinant Hsp90 ATP-binding domain assay relies on competitive inhibition of bis-ANS competes with ATP for the ATPase domain and emits fluorescence upon binding at the hydrophobic pocket (Wassenberg et al., 2000). This suggests that the hit compounds identified here compete for the ATP-binding domain. While the full binding mode has not been fully elucidated for these hits, the Hsp90 ATP-binding domain contains several basic and hydrophobic residues that are characteristic of protein binding pockets that bind structurally diverse compounds (Dutta and Inouye, 2000).

Only 19 out of the 43 compounds inhibited bis-ANS binding of the EhHsp90 ATP binding domain and 43 of the 43 compounds inhibited the HsHsp90 ATP binding domain. This result suggests that there is sufficient biochemical diversity in this pocket to allow for ortholog selectivity of some of these compounds. This hypothesis was further tested by the site directed mutagenesis study which showed selective binding of most of the 43 drugs tested upon switch of one of the three unique residues in each of the binding pockets of EhHsp90, PfHsp90, and HsHsp90. This experiment showed that sufficient structural diversity in the ATP-binding pocket of Hsp90 allows for ortholog selectivity of Hsp90 through manipulation of the architecture of EhHsp90, malaria and human Hsp90 by mutagenesis.

The 19 EhHsp90 hits were further tested against live *E. histolytica* trophozoites. Five of the 19 compounds exhibited inhibitory activity *in vitro*. The other 14 compounds may not have been able to penetrate the plasma membrane of the trophozoites *in vitro* or otherwise access EhHsp90. The five inhibitors of EhHsp90 with promising activity against the parasite were rifabutin, rutilantin, cetylpyridinium chloride, pararosaniline pamoate, and gentian violet. Rifabutin is a rifamycin-class antibiotic with an ansamycin moiety (O'Brien et al., 1987, 1990). Ansamycin inhibitors such as geldanamycin are well characterized inhibitors of the chaperone activity of Hsp90 (Bohen, 1998; Onuoha et al., 2007; Porter et al., 2009). Both rifamycin and rifabutin have been used widely against mycobacteria (O'Brien et al., 1987, 1990), but there is no report of their use against amebiasis. Other studies have suggested that rifabutin is effective against cryptosporidiosis, another intestinal parasitic infectious disease (Holmberg et al., 1998; Fichtenbaum et al., 2000). The antibiotic rutilantin has antiphage and antiviral activity (Asheshov and Gordon, 1961; Hume et al., 1965). Cetylpyridinium chloride is an antiseptic compound that is used in mouthwash and other mouth/throat care products (Sheen et al., 2003; Herrera et al., 2005; Gunsolley, 2008; Herrera, 2009; Rioboo et al., 2011). Potent activity has also been reported against a fungal pathogen: *Candida albicans* (Jones et al., 1995). Pararosaniline pamoate has already been used as an antiparasitic drug against schistosomiasis in the Philippines (Pesigan et al., 1967). This compound has shown anti-Hsp90 activity in another high throughput screen as well (United States Patent Application 20110263693). Gentian violet is also known as crystal violet and is one of the constituents of the Gram stain used to visualize bacteria (Berberovic, 1953; Saji et al., 1994). 1% solution of gentian violet is reported as a treatment for *C. albicans* infections (Traboulsi et al., 2011). Gentian violet and pararosaniline pamoate are structurally related molecules (triarylmethanes group). Considering this previous literature and actual usage, these drugs come with known pharmacokinetic, pharmacodynamics, and safety profiles that allow bypassing of the initial evaluation of bioavailability, metabolic stability, adsorption, and excretion.

## Conclusion

ATP is required for client protein and co-chaperone binding with Hsp90 (Prodromou et al., 1997). Therefore, inhibition of the Hsp90 ATP binding is lethal in all higher organisms studied to date and may prove effective in the treatment of amebiasis. The efficacy and safety of these candidate drugs needs to be reassessed in animal models. Even if the repurposed drug candidates reported here do not turn out to be optimal antiamebic agents, they provide a starting point for further structure-based drug design. Novel drugs for amebiasis as well as other neglected tropical diseases are urgently needed and Hsp90 provides a radically new target whereby resistance can be circumvented. The long term goal of these preliminary studies is co-formulation of drug combinations including Hsp90 inhibitors.

### Conflict of interest statement

The authors declare that the research was conducted in the absence of any commercial or financial relationships that could be construed as a potential conflict of interest.

## Supplementary material

Supplimentary File 1 **The list of chemical name abbreviations used throughout the manuscript**.

Supplimentary File 2 **List of primers used for site-directed mutagenesis**.
