# Supplementary material for: Heat shock protein 90 inhibitors repurposed against Entamoeba histolytica
Source: Front Microbiol. 2015 Apr 28;6:368. doi: 10.3389/fmicb.2015.00368 (PMC4429810; doi:10.3389/fmicb.2015.00368)
Supplement: Supplimentary File 1 — The list of chemical name abbreviations used throughout the manuscript. [file DataSheet1.PDF]

ACF	Acriflavinium hydrochloride
API	Apigenin
APSA	3-Amino-1-propanesulfonic acid sodium
BRB	Bilirubin
CHL	Chloranil
CLO	Clofazimine
CMDN	Chlormadinone acetate
CPC	Cetylpyridinium chloride
CPH	Chlorophyllide
CUR	Curcumin
DAU	Daunorubicin hydrochloride
DDB	4,4.-Dimethoxydalbergione
ELL	Ellipticine
EMO	Emodin
EPG	Epigallocatechin 3,5-digallate
ETH	Ethaverine hydrochloride
FPC	Fumarprotocetraric acid
GVI	Gentian Violet
GW 5074	GW 5074
HDT	12a-Hydroxy-9-demethylmunduserone-8-carboxylate
HYC	Hycanthone
KAR	Karanjin
MAN	N-methylanthranilic acidN-METHYLANTHRANILIC ACID
MDAP	4-methyldaphnetin
MND	Menadione
MTX	Mitoxantrone dihydrochloride
ORS	Orsellinic acid
PHD	Phenindione
PHH	Phenazopyridine hydrochloride
PMC	Palmatine chloride
PSP	Pararosaniline pamoate
PUR	Purpurogallin
QNC	Quinacrine dihydrochloride
RHT	Rhetsinine
RIF	Rifaximin
ROS	Rosolic Acid
RSQ	Retusoquinone
RUT	Rutilantinone
SU 4312	SU 4312
SU 6656	SU 6656
TYR	Tyramine
VPF	Verteporfin
WB 64	WB 64 (Malachite Green)
