# Supplementary material for: Heat shock protein 90 inhibitors repurposed against Entamoeba histolytica
Source: Front Microbiol. 2015 Apr 28;6:368. doi: 10.3389/fmicb.2015.00368 (PMC4429810; doi:10.3389/fmicb.2015.00368)
Supplement: Supplimentary File 2 — List of primers used for site-directed mutagenesis. [file DataSheet2.PDF]

PfHsp90 (R98K)
ORIGINAL SEQUENCE: GGTACTATTGCA**AGA**TCAGGAACCAAA
Forward primer: 5'- GGT ACT ATT GCA **AAA** TCA GGA ACC AAA -3'
COMPLEMENT: 5'- TTT GGT TCC TGA TTT TGC AAT AGT ACC -3'

PfHsp90 (A38S)
ORIGINAL SEQUENCE: TTGATTAGTAAT**GCT**AGTGATGCCTTA
Forward primer: 5'- TTG ATT AGT AAT **TCT** AGT GAT GCC TTA -3'
COMPLEMENT: 5'- TAA GGC ATC ACT AGA ATT ACT AAT CAA -3'

PfHsp90 (A38C)
ORIGINAL SEQUENCE: TTG ATT AGT AAT **GCT** AGT GAT GCC TTA
Forward primer: 5'- TTG ATT AGT AAT **TGT** AGT GAT GCC TTA -3'
COMPLEMENT: 5'- TAA GGC ATC ACT ACA ATT ACT AAT CAA -3'

EhHsp90 (R109K)
ORIGINAL SEQUENCE: GGTACTATTGCA**AGA**TCAGGAACAAAG
Forward primer: 5'- GGT ACT ATT GCA **AAA** TCA GGA ACA AAG -3'
COMPLEMENT: 5'- CTT TGT TCC TGA TTT TGC AAT AGT ACC -3'
MELT TEMP: 54.8 ºC

EhHsp90 (C49S)
ORIGINAL SEQUENCE: TTA ATT TCA AAT **TGT** TCA GAT GCA CTT
Forward primer: 5'- TTA ATT TCA AAT **AGT** TCA GAT GCA -3'
COMPLEMENT: 5'- TGC ATC TGA ACT ATT TGA AAT TAA -3'

EhHsp90 (C103N)
ORIGINAL SEQUENCE: ATT AAT **TGT** CTT GGT ACT ATT GCA
SEQUENCE: 5'- ATT AAT **AAC** AAT CTT GGT ACT ATT GCA -3'
COMPLEMENT: 5'- TGC AAT AGT ACC AAG ATT GTT ATT AAT -3'

EhHsp90 (C49A)
ORIGINAL SEQUENCE: TTA ATT TCA AAT **TGT** TCA GAT GCA CTT
SEQUENCE: 5'- TTA ATT TCA AAT **GCT** TCA GAT GCA CTT -3'
COMPLEMENT: 5'- AAG TGC ATC TGA AGC ATT TGA AAT TAA -3'

HsHsp90 (K112R)
ORIGINAL SEQUENCE: CTT GGT ACT ATC GCC **AAG** TCT GGG ACC
Forward primer: 5'- CTT GGT ACT ATC GCC **AGG** TCT GGG ACC -3'
COMPLEMENT: 5'- GGT CCC AGA CCT GGC GAT AGT ACC AAG -3'

HsHsp90 (S52C)
ORIGINAL SEQUENCE: CTC ATT TCA AAT **TCA** TCA GAT GCA TTG GAC
Forward primer: 5'- CTC ATT TCA AAT **TGT** TCA GAT GCA TTG GAC -3'
COMPLEMENT: 5'- GTC CAA TGC ATC TGA ACA ATT TGA AAT GAG -3'

HsHsp90 (N106C)
ORIGINAL SEQUENCE: GAC TTG ATC AAT **<u>AAC</u>** CTT GGT ACT
Forward primer: 5'- GAC TTG ATC AAT **<u>TGC</u>** CTT GGT ACT ATC -3'
COMPLEMENT:5'- GAT AGT ACC AAG GCA ATT GAT CAA GTC -3'

HsHsp90 (S52A)
ORIGINAL SEQUENCE: CTC ATT TCA AAT **<u>TCA</u>** TCA GAT GCA TTG GAC
Forward primer: 5'- AGA GAG CTC ATT TCA AAT **<u>GCA</u>** TCA GAT -3
'COMPLEMENT: 5'- ATC TGA TGC ATT TGA AAT GAG CTC TCT -3'
